# Transforming your daily work into scholarship: Tips for a busy clinician-scholar

**DOI:** 10.15694/mep.2019.000210.1

**Published:** 2019-11-20

**Authors:** Sarina Schrager, Susan Pollart, Elizabeth Sadowski

**Affiliations:** 1 University of Wisconsin; 2 University of Virginia

**Keywords:** Faculty development, scholarly activity

## Abstract

This article was migrated. The article was marked as recommended.

Academic faculty are pulled in multiple directions during their daily work life. Clinical issues, teaching responsibilities, and administrative duties all take up large amounts of time. At the same time, most faculty have requirements for scholarly activities for promotions. This paper discusses ways that individuals can use much of the work that they do in all of the realms of their jobs and turn it into scholarly projects. By focusing the content of their work, planning ahead, and making everything count twice, faculty can be successful in developing scholarship using their daily work.

## Introduction

Academic faculty are required to demonstrate a regional or national reputation for promotion through development of scholarly products. Scholarly products include items such as presentations, publications, abstracts, digital content, and published curricula. Disseminating work, either via traditional scholarly outlets or by capitalizing on new opportunities for information sharing, (i.e. social media) is essential for moving the discipline forward and allowing material to be available broadly.

Boyer’s classification of scholarship supports the many roles a clinician scholar fills throughout their career ( Boyer, 1990). Traditional academic medicine encompasses four pillars: clinical care, teaching, research, and administration, however outreach, policy, informatics, and quality initiatives have become ancillary paths a clinician can pursue. To be considered scholarship, the work needs to be clearly documented, peer-reviewed, and disseminated to the public ( Boyer, 1990). Please see Table 1.

**Table 1.** Boyer’s classification of scholarship

Boyer’s classification of scholarship	Description
Discovery	Investigation of new techniques, therapies, and processes (clinical care, teaching, informatics, quality improvement)
Integration	New interpretations of established evidence (clinical care, teaching, quality improvement, informatics)
Application	Sharing established knowledge outside of the academic community (clinical care, administration, teaching, outreach, policy, informatics)
Teaching	The study of how learners acquire and how teachers present new knowledge. (teaching, outreach, quality improvement, informatics)

## Translating daily activity into scholarship

We suggest the following five strategies to turn the daily work of the academic physician into scholarship.

1. Conduct a survey of your daily work.

2. Keep all of your work close to home, related to one or two themes.

3. Plan ahead.

4. Make everything count twice.

5. Use social media wisely to extend the impact of your work.

## Conduct a survey of your daily work

In a routine day, academic faculty physicians engage in many different work tasks: Morning rounds, clinic, committee meetings over lunch, academic time for reviewing data in the afternoon before medical student teaching, and community outreach on a new hospital service at night. Even “non-academic” activities can help increase expertise on a topic and you can begin to transform your everyday work in that area by spending time on the same topic in different venues. For example, increasing referrals of patients with a specific disease or for a specific procedure will result in more experience in this area. This in turn will allow you to sign up for a committee focused on the topic, give a talk and then write up your lecture or your experience with the topic in a specialty journal. Through this type of approach, you begin to “transform” your daily work into scholarly products.

## Keep all your work close to home, related to one or two themes

In the early stages of a career, exploration of career paths and options is expected and should naturally lead to a period of deep engagement where career goals are defined, developmental needs identified, potential barriers are elucidated, and milestones are set ( Viggiano, 2009). During this deeper period of engagement, usually 3 or 4 years into a career, faculty members should move beyond a broad exploration of potential career paths and academic foci and begin to relate all work to one or two themes. A concentration on these themes in all aspects of faculty life allows greater impact on the faculty member’s area of interest and expertise. For example, a faculty member who chooses a specific area of clinical focus may impact the clinical mission by extending this service to a new (or underserved) population. Expertise in this clinical area may open opportunities for participation in clinical trials or collaborative research projects about various aspects of this clinical work. This same faculty member may develop a curriculum related to the clinical focus that spans the population of trainees served at their institution from medical students to physician colleagues. He/she may volunteer to provide community education on this same area and may provide service on professional organizations’ committees or workgroups. On these committees or workgroups, the faculty member will have opportunities to build a reputation as an expert and to meet colleagues who can serve as collaborators, mentors, sponsors, and referees for academic advancement.

## Plan ahead

When you start a new project, volunteer for a new committee, or commit to giving a talk, think about how this activity can be turned into scholarship. Start every project with the question, “What is my outcome?” and use the information to design a way to answer the question (Britt).Think about doing a pre-test or survey to document the issue or problem to be addressed. That way, you are able to measure outcomes from your work. Plan for dissemination of your work before you even get started. How do you want to disseminate it? Who is the audience? Who would be interested in working on with you?

Previous work has discussed methods to “transform” a presentation into a publication, which is an example of taking work in one area (teaching) and making it into another (writing) ( Schrager, 2010). The process is predicated on the idea that you think about writing about the same topic that your presentation is covering, before you start preparing the talk. You can then use the same literature review, with careful notes, that you use for the presentation to subsequently write a paper.

## Make everything count twice

Keeping an open mind and being flexible about activities may also help with taking advantage of opportunities that come up and making things count twice ( Britt, n.d.). Dr. Rebecca Britt, in her podcast, “Making things count twice”, tells a story of teaching the skill of putting in a central line through a simulation lab. She had been looking for a better way to teach residents to insert central lines (rather than learning “live” on patients) and so she developed a curriculum to teach the skill via a simulated patient lab. Dr. Britt worked with a team and published several articles about the process ( Britt, n.d.). This process took more time and energy than simply teaching the resident a new procedure, but turned a clinical teaching experience into scholarship.

In order to optimize time, it is prudent for faculty to devote some of their clinical, teaching and administrative time to developing a scholarly product ( Schrager, 2016; Vicens, 2009). Faculty members can design an innovative course or a novel method for evaluating students. Both of these activities may be appropriate to write about in an education journal( Vicens, 2009). Students and residents may also provide valuable feedback on research ideas and often have energy and enthusiasm that is useful to develop new projects and help the current project progress.

Additional examples

• You start work on a committee that was formed to improve the care of adults with uncontrolled diabetes at your clinic. The committee discusses introducing group visits with a dietician and a fitness instructor. You think about how this could turn into a scholarly project. Before the group visits start, you gather data about BMI and hemoglobin A1C levels among your patients. You take notes about the barriers and difficulties in scheduling these visits and ways that the clinic tries to increase attendance. After 6 months, you have a cohort of 20 people who completed the project. You gather comparison data. You present this data at a primary care conference in your institution, then submit an abstract to a regional conference about models of care, and write a brief report about your findings that you submit for publication. If you had positive findings, you apply for a grant to support a larger program, maybe even doing a randomized controlled trial.
• You are working with a resident in the clinic and see a patient with an unusual clinical presentation. After doing a brief literature review while you are discussing this patient, you determine that there are no case reports of the particular clinical presentation. You enlist the resident (who is required to complete a scholarly project during residency) to do a more extensive literature review and write a case report with a literature review together. You also present your case together at a department wide case conference or grand rounds.
• You decide to develop a new curriculum to teach about implicit bias to first year medical students. You start thinking about publication at the beginning of the process, write clear learning objectives, develop multiple methods of evaluation and outcome measures that correspond to the learning objectives, make sure your evaluation measures have reliability and validity, and then decide which part of your curriculum you want to publish ( Kern, 2005). You submit the curriculum and evaluations from the learners for publication in MedEd Portal (or another peer reviewed electronic resource) because of the novelty of the curriculum. You may also consider starting the process with a pre-test and then do a post-test 6 months after the course to determine whether the course had a lasting impact on the students and publish these data.

Please see Tables 2, 3, and 4 which describe specific suggestions for transforming clinical, teaching, and administrative work into scholarship.

**Table 2.** Transforming clinical work into scholarship: ( Schrager and Sadowski, 2016)

• Write a case report about an interesting patient-rare disease, rare presentation of a common disease, common disease in a different patient population, new treatment for a common condition. • Review clinic data and present or write about a trend in patients with a certain diagnosis (new presentation, new treatment, or uncommon characteristics). For example, you see 3 women in their 30s with shingles. Is shingles becoming more common in young women? Can you ask for clinic data looking at ages and genders of all of the people who have presented with shingles in the last year? • Describe an innovative method for treating a common disorder that has worked in your practice • Write about a novel protocol that you developed in your office to treat people with XX disease. • Develop a method to treat pain during a specific procedure and consider doing a study about whether it is better than previous treatment.

**Table 3.** Transforming administrative work into scholarship: ( Schrager and Sadowski, 2016)

• Measure outcomes from a QI project • Write a narrative essay about the process of a committee meeting, a personal evolution in beliefs or behavior, or a commentary about the actual committee • Present a talk about specific methods for increased productivity or effectiveness • Write a position paper about a particular topic in health care administration • Volunteer to be a chair of a committee and use specific leadership skills. Then develop a talk for your institution about leadership skills. Submit the talk to a regional or national meeting.

**Table 4.** Transforming teaching into scholarship: ( Schrager and Sadowski, 2016)

• Experiment with different teaching methods to present the same information. Then do a post-test and compare results. • Write a paper about student’s learning styles and how you adapted your teaching to meet their needs. • Plan to write a paper about a talk that you are giving-keep track of all the references, take good notes in powerpoint, and revise the paper based on feedback you get from your talk ( Schrager, 2010). • Develop a curriculum that is innovative and new, and plan to publish it from the beginning ( Viggiano, 2009). • Develop a model to teach a specific procedure and evaluate if it is effective to teach students or residents.

## Use social media wisely

Social media provides new ways for faculty in academic medicine to connect personally and professionally and can be used effectively to learn, network, seek (and provide) mentorship, share academic accomplishments (including publications), and advance the faculty members professional reputation (Jaime, 2018). Twitter-based chats and journal clubs allow faculty members to develop learning communities that address a wide variety of topics. Social media platforms can also extend the formal learning opportunities by providing informal learning networks to complement formal supports and extend the learning environment ( Rehm, 2016).

Conference specific hashtags are now widely used and allow both meeting attendees and non-attendees to participate in conversations around cutting edge ideas and concepts that are showcased at national meetings. Posting and providing links to publications on social media platforms can greatly extend the impact of a publication. While the metrics around this type of dissemination of published work are not yet clearly defined, there is no doubt that multiple modalities to share original, peer-reviewed work will enhance the diffusion of new ideas as well as the reputation of its authors ( Sherbino, 2015; Chan, 2018).

## Conclusion

Aligning daily activities with one or two main areas of expertise can help the busy clinician-scholar use even non-academic time to work on scholarly pursuits. Traditional methods for scholarship dissemination can be supplemented with new modalities that have proven highly useful. With some foresight and organization, faculty physicians may be able to use much of their daily workload as a basis for scholarly activity.

## Take Home Messages

• Busy clinician educators can increase their scholarly activity by focusing on one or two themes in their scholarship, planning ahead and using the same activities in several different venues.
• Social media will help broaden the reach of scholarship.

## Notes On Contributors

Dr. Schrager is a Professor in the University of Wisconsin Department of Family Medicine and Community Health. She is the director of promotions and mentoring for the department and also is the director of the academic fellowship.

Dr. Pollart is the senior associate dean for faculty affairs and faculty development at UVA School of Medicine. She is also Walter M. Seward Professor of Family Medicine and interim chair of the department.

Dr. Sadowski is a Professor in the University of Wisconsin Department of Radiology and the Director of the Career Paths Faculty Development workshops.
