# Supplementary material for: Transforming your daily work into scholarship: Tips for a busy clinician-scholar
Source: MedEdPublish (2016). 2019 Nov 20;8:210. [Version 1] doi: 10.15694/mep.2019.000210.1 (PMC10712472; doi:10.15694/mep.2019.000210.1)
Supplement: Peer review history

# Reviewer report: Reviewer response for version 1

**DOI:** 10.21956/mep.19910.r30161

**Published:** 2019-12-02

**Authors:** P Ravi Shankar

**Affiliations:** 1 American International Medical University

This review has been migrated. The reviewer awarded 4 stars out of 5 I read with great interest this article on transforming everyday activities into scholarship. The suggestions by the authors are practical and feasible. When I was starting my academic career a senior faculty member told me that most activities and interventions should result in a paper and should be planned and designed as such. A pre-post measurement will be quite useful in this regard. Tables 2, 3 and 4 will be of special interest to readers. There is an ongoing interest in the areas of interprofessional collaboration and education which can be transformed into scholarship. Joining a community of practice can help clinicians convert more of their daily activities into scholarship. The FAIMER fellowships have been partly successful in creating physician scholars in South Asia though more basic science faculty join the fellowship compared to clinical faculty. The article will be of interest to all medical and health educators.

Reviewer Expertise:

NA

No decision status is available

# Reviewer report: Reviewer response for version 1

**DOI:** 10.21956/mep.19910.r30162

**Published:** 2019-11-24

**Authors:** Richard Hays

**Affiliations:** 1 James Cook University

This review has been migrated. The reviewer awarded 4 stars out of 5 Thanks for this interesting paper which addresses an issue of great importance to education scholars, given that rewards for scholarly activity are weighted towards traditional clinical and foundation science research. The junction between these forms of academic endeavour is where my career has sat for many years. I was hired to promote primary care research, and yet medical education was my passion. Luckily, the definition of the former is quite broad. I can suggest another couple of tips. 1. Designing, implementing and evaluating education interventions may be helped by funding/resource allocation/ethics exemptions, as most people are interested in a good outcome. Therefore, design well, based on the available literature and evidence about experience elsewhere, implement as well as possible (bring the team with you) and then design high quality evaluation. Finally, make sure the outcomes are considered for publication. 2. Try not to allow scholarly activity 'çompete' with your other roles'by accepting roles that include stewardship of educational development. Some research granting bodies will consider funding this as they want a good outcome that included translation into practice. In other words, make educational scholarship an explicit part of your role that is included in annual appraisal processes. In the early part of my career I managed to achieve about 30% of my publications in education scholarship by following these steps.

Reviewer Expertise:

NA

No decision status is available

# Reviewer report: Reviewer response for version 1

**DOI:** 10.21956/mep.19910.r30158

**Published:** 2019-11-23

**Authors:** John Cookson

**Affiliations:** 1 University of Worcester

This review has been migrated. The reviewer awarded 4 stars out of 5 I enjoyed reading this and I'm sure it will be helpful to younger academic clinicians. On reflection, there will still be problems if promotion committees use publication in high impact journals as the only currency.

Reviewer Expertise:

NA

No decision status is available

# Reviewer report: Reviewer response for version 1

**DOI:** 10.21956/mep.19910.r30159

**Published:** 2019-11-20

**Authors:** Subha Ramani

**Affiliations:** 1 Harvard Medical School, Brigham and Women's Hospital

This review has been migrated. The reviewer awarded 4 stars out of 5 This is a well written, easy to read article for clinical educators who wish to enhance their scholarship and position themselves for academic advancement. The strategies are practical and relevant to all clinicians who have roles in education but find it challenging to convert their educational products into scholarly output. One area that is being emphasized more in faculty development is the concept of 'communities of practice'. Strategies to join a community are not included in this article besides social media. I would like to make a strong recommendation to all educators aiming for scholarship. There is only so much one can do alone.Mentoring is mentioned in passing and I would like to highlight its importance in academic advancement.Lastly, a few choice references should be looked at- papers by Simpson et al and Glassick.This paper will be useful to novice educators who are considering engaging in scholarship.

Reviewer Expertise:

NA

No decision status is available

# Reviewer report: Reviewer response for version 1

**DOI:** 10.21956/mep.19910.r30160

**Published:** 2019-11-20

**Authors:** Felix Silwimba

**Affiliations:** 1 University of Lusaka

This review has been migrated. The reviewer awarded 5 stars out of 5 this write and guide to medical educators is clear and practical. I have personally learnt something valuable to put to good use.

Reviewer Expertise:

NA

No decision status is available
